# Evaluation of Four Commercial Vaccines for the Protection of Piglets against the Highly Pathogenic Porcine Reproductive and Respiratory Syndrome Virus (hp-PRRSV) QH-08 Strain

**DOI:** 10.3390/vaccines9091020

**Published:** 2021-09-14

**Authors:** Yaozhong Ding, Ashenafi Kiros Wubshet, Xiaolong Ding, Zhongwang Zhang, Qian Li, Junfei Dai, Qian Hou, Yonghao Hu, Jie Zhang

**Affiliations:** 1 State Key Laboratory of Veterinary Etiological Biology, National Foot-and-Mouth Disease Reference Laboratory, Lanzhou Veterinary Research Institute, Chinese Academy of Agricultural Sciences, Lanzhou 730046, China; nafikw@gmail.com (A.K.W.); dyz1953@126.com (X.D.); zhangzhongwang@caas.cn (Z.Z.); qanli985@163.com (Q.L.); aixinjueluofei@hotmail.com (J.D.); qianh910@163.com (Q.H.); 2 College of Veterinary Medicine, Gansu Agricultural University, Lanzhou 730070, China; yhh0817@126.com; 3 Department of Basic and Diagnostic Sciences, College of Veterinary Science, Mekelle University, Mekelle 280, Ethiopia; 4 Hebei Normal University of Science and Technology, Qinhuangdao 066004, China

**Keywords:** PRRSV, phylogenetic analysis, vaccine efficacy, vaccine design ORF1a-1bs of PRRSV, piglets

## Abstract

Vaccination is the best way to prevent economic losses from highly pathogenic porcine reproductive and respiratory syndrome virus (hp-PRRSV) disease. However, the commercially available vaccines need to periodically evaluate their efficacy against infections caused by new hp-PRRSV variants. Therefore, the objective of this study was to evaluate the efficacy of four (two modified live vaccines (MLV) and two inactivated) PRRSV commercial vaccines in piglets challenged with QH-08 and to estimate the genetic distance of the vaccine strains from recently isolated (QH-08) filed strain. Randomly, piglets (n = 5) allocated in groups 1–4 were immunized with Ingelvac PRRS MLV, CH-1a, JXA1, and JXA1-RMLV vaccines, whereas the infected and non-infected control piglets in groups 5 and 6 (n = 3), respectively, were subjected to PBS. Results indicated that JXA1 and JXA1-R MLV vaccines showed complete protection, but Ingelvac PRRS MLV and CH-1α vaccines revealed partial protection against the QH-08 PRRSV challenge. Similarly, vaccinated and challenged pigs showed lower macroscopic and microscopic lesions than the pigs in group 5. Our findings demonstrated a new insight that the variation in ORF1a and 1b coding sequence could significantly affect PRRSV vaccines efficacy. In conclusion, QH-08 is a good candidate for the design and development of an innovative PRRSV vaccine that ultimately helps in the control and prevention strategies.

## 1. Introduction

Porcine reproductive and respiratory syndrome (PRRS) is a highly contagious swine disease, characterized by acute respiratory distress in piglets and reproductive failure in sows leading to tremendous economic losses worldwide [1]. The etiological agent porcine reproductive and respiratory syndrome virus (PRRSV) is an enveloped single-positive-stranded RNA virus of the family *Arteriviridae* [2,3,4]. The virus has an approximately 15 kb length genome that possesses nine or ten overlapping open reading frames (ORF): ORF 1a-1b, ORFs 2–7 (viral structural proteins GP2, E, GP3, GP4, ORF5a, ORF5, M, and N) [5]. The PRRSV virion possesses a nucleocapsid containing the viral genome covered by a lipid envelope encompassing the structural protein E, GP2, GP3, GP4, ORF5a protein, GP5, and M [5]. Historically, PRRSV comprised type 1 (PRRSV-1) and type 2 (PRRSV-2); recently, PRRSV-1 was taxonomically classified into the species *Betaarterivirus suid 1* and PRRSV-2 into the species *Betaarterivirus suid 2*.PRRSV genotype types 1 and 2 [6,7], into biologically new hierarchical classification, as *Betaarterivirus suid 1* species and *Betaarterivirus suid 2* species, respectively [8].

Based on the degree of pathogenicity, PRRSV is divided into classical PRRSV (C-PRRSV), special mutant PRRSV (M-PRRSV), and highly pathogenic PRRSV (hp-PRRSV) strains [9]. Out of the three types, a highly pathogenic PRRSV (hp-PRRSV) outbreak emerged in China for the first time in 2006 and caused substantial economic losses [10]. Although the Betaarterivirus suid 1 species (European genotype) and (North American genotype) Betaarterivirus suid 2 species synchronously occurred and share similar clinical signs, their genetic and antigenic characteristics are remarkably different [11,12]. 

Vaccination is always the primary option to control and eradicate deadly diseases. Still, some inactivated vaccines do not often protect the animals against PRRSV challenges when major antigenic shifts or novel virus subtypes appear [13,14,15,16]. The molecular mechanism of protecting the PRRS vaccine is due to the expression of ORF1a and 1b of PRRSV. Additionally, genes profiled in ORF2 to ORF7 also play a significant role in this regard. Vaccines that can provide cross-protection are urgently needed. Thus, significant research efforts have been made to verify the protection spectrum that commercial or experimental vaccines can afford. To the best of our knowledge, there is no report stated so far on the considerable influence in the protection potency of vaccines due to the ORF1a and 1b PRRSV genes coding sequence differences. 

To further improve the performance of the commercial vaccines and depth of the immune responses, our study aims to evaluate the efficacy of four (two modified live vaccines (MLV) and two inactivated) PRRSV commercial vaccines in piglets challenged with QH-08 PRRSV isolate and to estimate the genetic distance of the vaccine strains from recently isolated (QH-08) field strain. Additionally, this study provides new insights and the first report on the significant importance of ORF1a and 1b genetic variation in the immunodynamics and efficacy of different kinds of PRRSV vaccines generated from the two species of PRRSV. 

## 2. Materials and Methods

### 2.1. PRRSV Strains Sequence Profiles from GenBank and Analysis 

The complete genome nucleotide sequence and amino acid sequences coding genes of ORF1a and 1b and ORF2-ORF7 of 12 Chinese and 21 foreign PRRSV strains (species 1 and 2) were obtained from GenBank with accession no listed in Table 1 and Table S1. The obtained nucleotide and amino acid sequences in this study were vaccine strains versus field strains originated from 14 countries from 1991 to 2018, particularly the genetic variation and phylogenetic relationships between the reference QH-08 field strain and the selected vaccine strains based on entire genome nucleotides and ORF1a and 1bs, ORF2-7 amino acid sequence (Table 1), with other strains originated from different countries (Figures S1–S3). All the sequences were aligned with the Clustal W method and analyzed using DNASTAR Lasergene (Inc. USA version 7) and MEGA 6.06. Furthermore, the percentages of nucleotide and amino acid sequence similarities were estimated by the pairwise distances method. The phylogenetic tree was constructed by neighbor-joining methods within MEGA 6.06 software suite [17] and bootstrap resampling used at 1000 replicates.

### 2.2. Cells, Virus Isolation, and Vaccines 

Type 2 PRRSV QH-08 strain (Gene Bank No: KU201579) was isolated in our laboratory from a herd of sow and piglets characterized by clinical respiratory signs with high morbidity and mortality. In vitro isolation methods were used in MARC-145 cells (derived from African green monkey kidney cells) to isolate the virus. Real-time polymerase chain reaction (RT-PCR) analysis was applied on the supernatants to confirm the growth of the virus. The presence of virus-infected cells in the 6-well plate was also determined using the plaque assays, qPCR, and indirect fluorescent antibody (I.F.A.) methods [3]. The QH-08 PPRSV strain was passed three times in MARC-145 cells used as a challenge virus during the experimental study on vaccine efficacy evaluation.

The four vaccines enrolled in this study are (1) modified live virus vaccine (MLV); (2) VR-2332 (Ingelvac PRRS MLV), obtained from Boehringer Ingelheim Vetmedica, Inc.; (3) CH-1R, JXA1; and (4) JXA1-R MLV vaccines obtained from Gansu Animal Health Company (Gansu, China). These vaccines were selected because of their frequent use in the control and prevention practices against PPRSV. Hence, we evaluated the capability of infection of QH-08 PRRSV isolate in the Ding repoted [4] which was 15,353 nucleotides (nt) in length including poly(A) tails. Meanwhile, this strain exist low recombination rates [18] which prevalent in Gansu and Qinghai Province

### 2.3. Animal Vaccine/Challenge Design

In this study, twenty-six 3-week-old young piglets (male, commercial breed, Kangle County Leixin Pig Breeding Co., Ltd., Gansu province) specifically free of PRRSV, PCV2, P.R.V., and CSFV infection were used as a respiratory disease model during the experimental period. Twenty of these piglets were randomly grouped into four distinct treatment groups (5 pigs/group). The other six were also randomly grouped into non-infected and infected control (3 pigs/group), and all were fed separately in different isolation rooms. 

Group 1 (Ingelvac PRRS MLV+QH-08 challenge), group 2 (CH-1α+QH-08 challenge), group 3 (JXA1+QH-08 challenge), and group 4 (JXA1-R MLV+QH-08 challenge) were vaccinated intramuscularly with a single dose as detailed in Table 2. According to the manufacturer’s manual, the amount of the virus in vaccine Ingelvac PRRS MLV, CH-1a, JXA1, and JXA1-R MLV is 10^5^ TCID50/mL, (≥2 × 10^7^ TCID50/mL), (≥2 × 10^7^ TCID50/mL), and (10^6^ TCID50/mL), respectively. 

Group 5 (QH-08-infected control) and group 6 (non-infected control) did not receive any of these four vaccines (Table 2). Twenty-eight days post-vaccination (dpv), which means 0-day post-challenge (dpc), the groups from 1 to 5 were infected with 2ml of QH-08 PRRSV strain (2 × 10^6^ TCID50/pig). Pigs in group 6 served as the non-infected control and were injected with 2 mL of PBS. All piglets were monitored daily for 13 days post-challenge (dpc), and at 14 dpc all piglets were euthanized and necropsied (Figure S1). The young piglets were sacrificed once the clinical signs became severe according to the ethical protocol and permit of the Biosecurity Committee of Lanzhou Veterinary Research Institute, Chinese Academy of Agricultural Sciences.

### 2.4. Clinical Examinations

The experimental animals were observed for any apparent changes in physical conditions and clinical respiratory disorders, such as behavior, cough, and breathing pattern. The body temperatures (°C) of all groups of animals were measured rectally every other day from the first day of vaccination to the last day of the post-challenge. The parameters were scored based on the Wei. et al. referencing scale, which ranges from 1 to 4 [19].

### 2.5. Serology

According to the manufacturer’s instruction manual, the sera were used to detect anti-PRRSV antibodies using the IDEXX PRRS X3 ELISA test kit (IDEXX Laboratories, Inc., Westbrook, ME, USA). The standardized positive (S/P) ratio value equal to or greater than 0.4 is considered as positive. As previously described by Thanawongnuwech et al. [16], the IPMA assay was used to confirm the presence of anti-PRRSV antibodies in serum samples.

Serum neutralization antibody (VN) assay was performed at 0 and 7 dpc using virus infection as previously described (Sirisereewan et al., 2017). Positive samples were considered to be positive for neutralizing antibodies (NAs) if the titer ≥ 1:2 (1log2). The presence of PRRSV was confirmed by IPMA assay as previously described [16].

### 2.6. RT-PCR for Antigen Detection

PRRSV RNA was extracted from both serum and fresh frozen lung tissues to detect viral genomic cDNA copy numbers. Viral RNA extraction was carried out using Takara MiniBEST RNA/DNA Extraction Kit (TaKaRa Bio Inc., Japan) following the company instructions. TaqMan^®^ probe-based real-time RT-PCR was used to quantify the copy number of viral RNA Primers, and the probe was synthesized as forward primer (Fp); 5-ATGATGRGCTGGCATTCT-3, the reverse primer (Rp); 5-ACACGGTCGCCCTAATTG-3, and the probe; 5-HEX-TGTGGTGATGGCACTGATTGACA-3. Furthermore, the total amount of RT-PCR mixture was (25 μL) composed of One-Step RT-PCR kit (Taqman probes, MyLab, China), 12.5 μL of 2× One-Step RT-PCR Enzyme Mix, 0.5 μL of each primer (10 μM) and probe (10 μM), 0.5 μL of RT-PCR Enzyme Mix, two μL of viral RNA, and 8.5 μL of RNase-free ddH_2_O. RT-PCR was conducted in the CFX96^TM^ real-time PCR System (BIO-RAD, Ltd., USA). The sequence analysis was used for detecting the virus RNA in sera and lung tissue coming from vaccination or challenge.

### 2.7. Statistical Analysis

Graph Pad Prism version 6.0 and Microsoft Excel were used to analyze the findings statistically. Accordingly, a two-sided *p* ≤ 0.05 was taken as the significant statistical variation between variables.

## 3. Results

### 3.1. Gene Identity and Phylogenetic Analysis

The amino acid sequence alignment results showed that the QH-08 isolate shared a higher average sequence similarity (97.9%) with JXA1 (Table 1). Furthermore, strains QH-08 have 96.3% homology with the ORF1a-1b of JXA1, but, which was observed 25.3 and 28.2% homology based on ORF1a-1b amino acid coding compared with CH-1a and VR2332 isolates, respectively. Based on coding sequences of ORF**_2-7_**, the QH-08 showed the least genetic variation among all vaccine strains.

The difference in sequence identity of ORF1a-1bs among the vaccine isolates with QH-08 isolate is a good implication for complete protection of JXA1-R MLV and JXA1 type 2 vaccines against the hp-PRRSV QH-08 challenge, unlike Ingelvac PRRS MLV and CH-1α vaccines.

**Figure 1:**
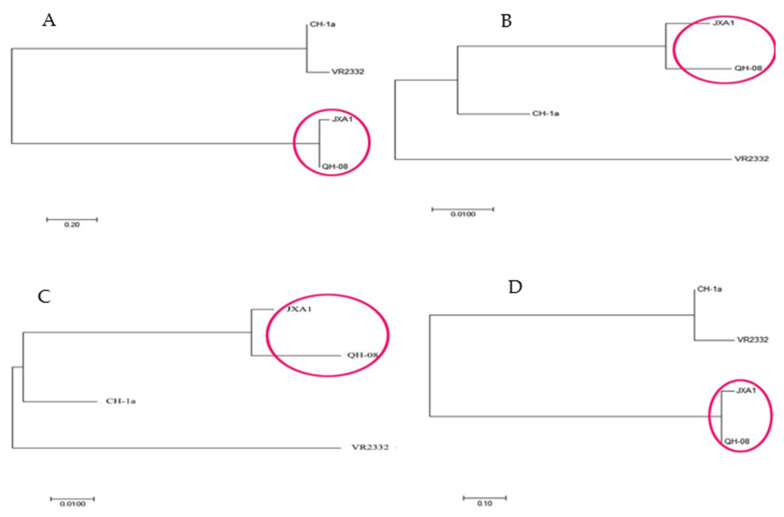
Phylogenetic comparison of the four PRRSV vaccines with reference filed strains (QH-08 isolate) based on amino acid sequences of (**A**) ORF1a and 1b, (**B**) ORF2–ORF7, (**C**) ORF5s, and (**D**) whole-genome sequence. The closely related strains are marked with the red color in a circle as indicated on the trees. Each bar represents substitutions per nucleotide position.

Briefly, we found that the percentage of ORF6 sequences similarity (92–98.9%) within the strains in the Chinese mainland showed distinctive gene conservation compared to the other genes, followed by ORF7, ORF4, ORF2, ORF3, and ORF5. ORF7, ORF2, and ORF5s genes showed insertion and deletion sites, such as Aomori10-5 in the JXA1 strain. Phylogenetic tree based on whole-genome sequence, ORF2–ORF7, and ORF1a-1bs, among the study vaccine strains indicated that JXA1 had a strong relationship with QH-08 isolate (Figure 1, red circle). We investigated that the gap in ORF1a-1b homology caused the sequence difference in the complete genome of PRRSV strains and may be related to the partial protection of the PRRS vaccines when challenged with some homologous virus.

High genetic diversity is a significant characteristic of PPRSV, the comparison of nt identity of whole-genome of the 33 species 1&2 based on QH-08 strains revealed that QH-08 had the minimum and maximum identity (33.7% and 97.2%, respectively) with SD-A19 and JXA1 (97.2%) in the Chinese reported sequence (Table S1). Similarly, the identity of 33 PPRSV isolates based on the whole nucleotide and/or amino acid sequence (aa) of ORF1a-1b and ORF2-7 is detailed in Table S1 and Figures S2–S4 by phylogenetic trees.

### 3.2. Clinical Observation

No adverse clinical symptoms or systemic effects, such as abnormal behavior or cough, were observed in vaccinated pigs.

**Figure 2:**
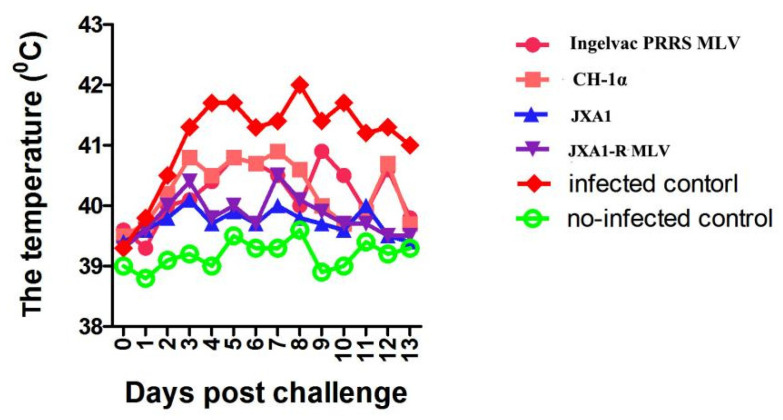
Comparison of the mean rectal temperature of pigs treated with different trials in the experiments. Each point represents the mean (±SD) generated from all of the piglets in each group after challenge. There was no significant difference in the rectal temperature between treatment and control groups compared to each other on days from 2 to 14 dpc (*p* < 0.05).

Post-challenge effect on body temperature of pigs in each group is shown in Figure 2. At 4-, 5-, 6-, 9-, and 10-days post-challenge, the rectal temperatures of piglets in group 4 which vaccinated with JXA1-R MLV were lower (*p* < 0.05) than of piglets in group 1 that were vaccinated with Ingelvac PRRS MLV.

The mean rectal temperature of piglets in JXA1+QH-08 was roughly lower than pigs in the vaccinated CH-1α+QH-08 challenge at 3, 4, 5, 7, 8, and 12 dpc and infected control at 2, 3, 4, 5, 7, 8, 10, and 13 dpc (Figure 2), compared with non-infected control which scored considerable body temperature. On the other hand, the mean rectal temperature of piglets in Ingelvac PRRS MLV+QH-08 showed no significant variation with pigs in the vaccinated CH-1α +QH-08 challenge.

**Figure 3:**
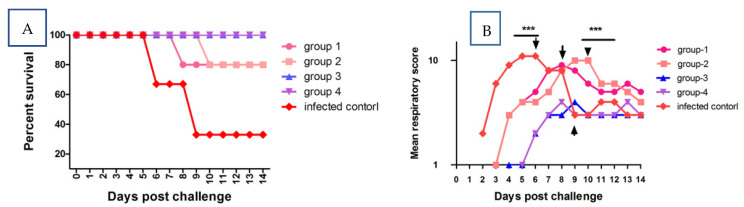
The survival rate and mean respiratory score of survived pigs. The points represent the mean (± SD) generated in all groups at the different time points after post-challenge. (**A**) This graph presents the time of death for the various groups by QH-08 PRRSV infection. (**B**) The piglets from groups 1–5 were infected with QH-08 PRRSV at 0 dpc pigs in group 1 to group 4 were significantly lower than group 5 pigs (infected control) at 2-5, 9 and 10 dpc (*p* < 0.001). *** *p* < 0.05.

The onset of an apparent clinical characteristic of the disease in the JXA1-R MLV+QH-08 and JXA1+QH-08 group of pigs was delayed several days, and no mortality occurred during the experimental periods (Figure 3A). Two pigs from infected control died at 6 (n = 1) and 9 (n = 2) dpc. Meanwhile, onr pig each from group 1 and group 2 died at 8 (n = 1) and 10 (n = 2) dpc, respectively.

Interestingly, the severity of the clinical signs was less in group 3 (JXA1+ QH-08) and group 4 (JXA1-R MLV+QH-08) pigs as compared to pigs in group 1 (Ingelvac PRRS MLV+ QH-08) and group 2 (CH-1α + QH-08). Mainly, pigs in group 3 did not show noticeable clinical signs such as the erythema of skin and shivering in the experimental periods. Moreover, the mean respiratory scores of pigs from group 1 to group 4 were significantly lower (*p* < 0.05) than infected control pigs at 2-5, 9, and 11 dpc (Figure 3B). However, the non-infected control maintained the expected temperatures and respiratory patterns throughout the experiment.

### 3.3. Detection of Virus RNA in Sera and Lung Tissue

Group 1–4 pigs have shown lower viral load in the serum from day 4 to 20 compared to infected control. Following the challenge, in infected control pigs, the viral titer detected on the second day after the challenge (10^6.8^ copies/mL) steadily increased on 9 dpc (10^9.3^ copies/mL) and then remained at a higher level until the end of the experiment.

**Figure 4:**
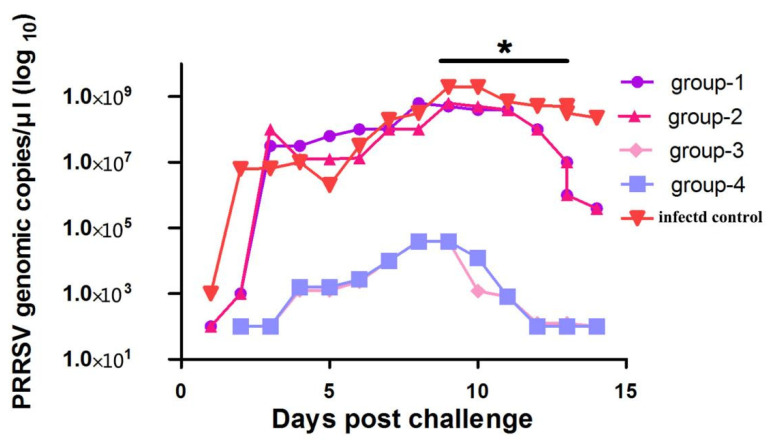
Viral titer in serum. The points represent the mean (± SD) generated in all groups at the different time points after post-challenge. The piglets from group 1, group 2, and group 5 show significantly higher viral titer than groups 3 and 4. * *p* < 0.001.

On the other hand, the piglets in group 1 had a high viral titer on the third day post-challenge (10^7.5^ copies/mL), which elevated on 8 dpc (10^8.8^ copies/mL) and then remained high until 10 dpc (Figure 4). Fourteen days after the challenge, the mean viral titer in group 1 was progressively reduced (RRRSV RNA: 10^5.6^ copies/mL) compared to the infected control pigs (*p* < 0.001) (Figure 4). Similarly, group 2 had a high viral titer on 4 ^th^ day dpc (10^7.1^ copies/mL), which peaked at 10^8.7^ copies/mL at the 9 dpc (Figure 4). The mean serum viral loads in both group 3 and group 4 were steadily reduced to 10^2.1^ copies/mL from 10^4.6^ copies/mL and were significantly lower as compared to the infected control (*p* < 0.001). The viral load in groups 3 and 4 remained low from the tenth dpc to the end of the experiment (Figure 4). Especially, the viral loads were significantly varied in groups 1, 2, and 3 at 3, 7, 10, and 14 dpc (Figure 4).

Macroscopic lung lesions were characterized by well-demarcated and consolidated areas with diffuse tan-brown discoloration observed in the middle, caudal, and accessory lobes. Pigs from group 1 to group 4 showed significantly fewer (*p* < 0.05) mean macroscopic lung lesions than pigs in infected control (Table 3). Microscopic lung lesions are characterized by thickening the alveolar septal with interstitial infiltration of macrophages and lymphocytes, type II pneumocyte hyperplasia, and the accumulation of normal and necrotic macrophages, specifically in alveolar spaces. The microscopic lesion is observed by hematoxylin and eosin staining (H&E). The mean microscopic lung lesion in pigs from group 1 to group 4 exhibited significantly lower lesions (*p* < 0.05) than pigs in infected control. Pigs in group 3 and group 4 also showed considerably lower (*p* < 0.05) mean microscopic lung lesion scores compared with group 1 and group 2 (Table 2). No macroscopic and microscopic lung lesions were observed in non-infected control.

### 3.4. Detection of Serum Antibodies against PRRSV

**Figure 5:**
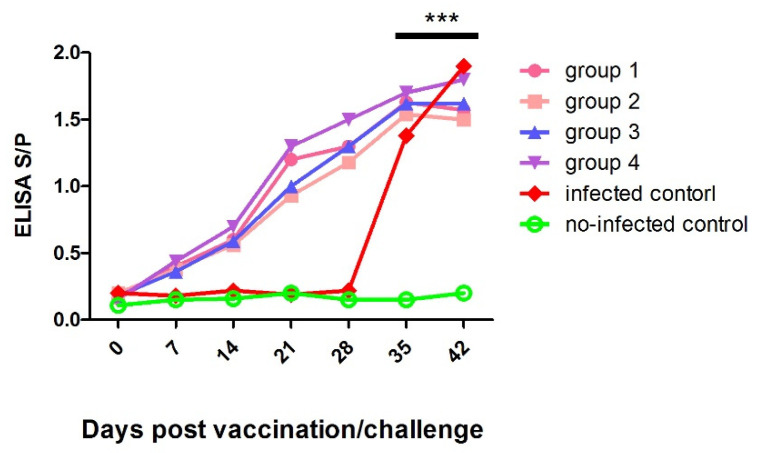
The average anti-PRRSV antibody levels in pigs were challenged at 28 days post-vaccination. The PRRSV-specific antibody development was monitored throughout the vaccination and challenging periods. In our experiment, the S/P ratios greater than 0.4 were considered positive. *** *p* < 0.001.

Briefly, after immunization, piglets from group 1 to group 4 had significantly higher (*p* < 0.001) anti-PRRSV antibodies than those of non-infected control from 14 to 28 dpv. Following the QH-08 challenge, the anti-PRRSV antibody titers in pigs in group 5 were higher than the pigs in group 6 at 4 and 7 dpc (Figure 5).

**Figure 6:**
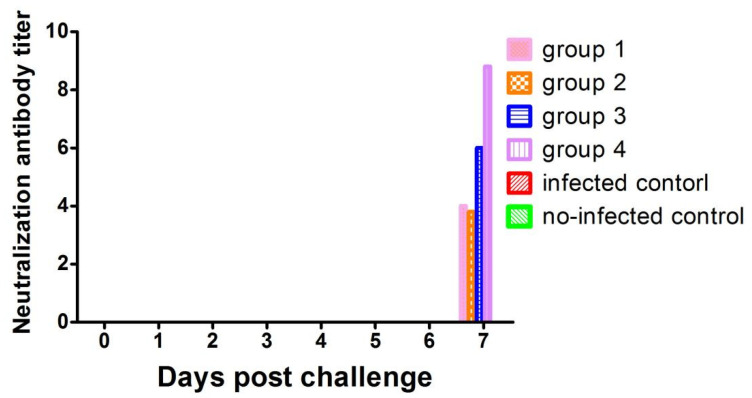
Serum neutralizing antibodies were detected using PRRSV infection of Marc-145 cells. The serum was considered neutralizing at four-fold or higher dilution in a positive response.

PRRSV-specific NA was not detected in all pigs on a challenging day (0 dpc). Interestingly, NA titers could be detected at 7 dp in most piglets from groups 1–4 and significantly increased compared to 0 dpc (Figure 6). Pigs in the negative control group had no neutralizing antibodies throughout this study.

## 4. Discussion

Porcine reproductive and respiratory syndrome (PRRS) is a highly infectious, rapidly transmissible, and economically important respiratory and reproductive viral disease of pigs of all ages. The PRRSV is a viral causative agent that remains a potential threat to swine production worldwide and causes enormous economic losses every year in endemic areas. Vaccination is an essential method to prevent, control, and further eradicate this viral infection. However, some reports show different types of commercial PRRSV vaccines lack a warranty for complete protection against new PRRSV subtypes [13,14,15].

In China, over 100 prevalent PRRSV-2 trains have been reported, as shown in [20], including four lineages: lineage 1, lineage 3, lineage 5, and lineage 8. Lineage 8 is predominant and includes classical PRRSV strains (CH-1a-like) prevalent before 2006 and HP-PRRSV-like strains prevalent after 2006. The lineage 1, also named as NADC30-like strains, has spread rapidly around the country since 2013 and show high pathogenicity comparable with HP-PRRSV-like. Since 2010, lineage 3 has been another newly emerged variant, which is mainly circulating in the south of China, including Jiangxi, Fujian, Guangdong, and Guangxi provinces. Although the lineage 5 (BJ-4-like/VR2332-like) appeared as early as 1996, it has always been non-pandemic in China, and the clinical detection rate is low. More than seven commercial PRRSV vaccines, including CH-1a/CH-1R, VR2332/Ingelvac PRRS MLV, R98/R98 MLV, JXA1/JXA1-R, TJ/TJM-F92, HuN4/HuN4-F112, GD/GDr180, etc., were used in China [20], but PRRS is still severe in the pig industry; especially the lineage 8 (HP-PRRSV-like) and lineage 1 (NADC30-like) have become the major epidemic strains, while lineage 8 (CH-1a-like) and lineage 5 are always endemic in China. The reasonable explanation for this is that HP-PRRSV-like and NADC30-like strains show high genetic variations and incidence of recombination compared with lineage 8 (CH-1a-like) and lineage 5 (the capacity of recombination of PRRSV-2 strains in China; see review: “The prevalent status and genetic diversity of porcine reproductive and respiratory syndrome virus in China: a molecular epidemiological perspective”). Meanwhile, we ensure that QH-08 strain exists in low recombination rates such as point deletions or insert rather than in the most genetically diverse regions (Figures S5–S10), but the rate of recombination of other strains increased dramatically from 2012 to 2015 [18]

These abovementioned characteristics probably made current vaccines ineffective and conferred them much easier to escape the immune surveillance. Thus, commercially available vaccines need to periodically evaluate for their efficacy against new hp-PRRSV variants infections [20,21,22].

The previous study asserted that the ORF5 sequence similarity between the PRRSV field and PRRSV vaccine strains provided better protective efficacy in the selected PRRSV vaccines [23]. However, vaccination with different PRRS vaccines did not provide significant differences for protective efficacy against the hp-PRRSV belonging to lineage 8 [13,14,15], indicating that having the same genetic lineage is not a reliable parameter to guarantee the vaccine protection.

In addition, gene sequence similarity analysis of ORF5 from hp-PRRSVs and classical type 2 PRRSV in China indicated higher homologousness (98.2–100%). Still, these vaccines work well for classical type 2 PRRSV provided limited protection against hp-PRRSVs. Despite the distinct differences in the percentage of the sequence identity in ORF 2–ORF 7 [14,16] (≥ 85%, Table 1), we hypothesized that the sequence identity percentage of the ORF1a-1bs of the PRRSV could affect the PRRS vaccines to have partial or complete protection to the homologous viruses challenges.

This study affirmed that the JXA1-R MLV and JXA1 type 2 vaccines provided more protection against the hp-PRRSV QH-08 challenge than Ingelvac PRRS MLV and CH-1α vaccines. The variation in protection capacity might come from the difference in sequence identity of ORF1a-1bs between the two strains and the virus in the challenge. Here, the sequence between JXA1 and QH-08 had a higher similarity (96.3%) than Ingelvac PRRS MLV and CH-1α (25.3–28.2%) (Table 1). Our study demonstrated that the Ingelvac PRRS MLV and CH-1α were less effective in protecting growing pigs against the QH-08 challenge, whereas JXA1-R MLV and JXA1 type 2 vaccines fully protected all pigs against the similar strain challenge caused by the identity of ORF1a-1bs.

On the other hand, our result revealed that the PRRSV-specific virus antibodies and serum NA of PRRSV-specific appeared after vaccination was challenged. This result is similar to previous studies [24], where the PRRSV vaccination-induced protection against homologous and heterologous challenge. Moreover, PRRSV induced neutralizing antibodies to seem associated with the neutralizing epitopes located in the first 60 amino acids of the GP5 gene [25], partially protecting PRRVs challenged [16].

Labarque G et al. [21] reported the results similar to the current findings; the commercial PRRS vaccines revealed a partial/complete protection to piglets against heterologous or homologous viruses [14,15,16]. Furthermore, we should concern that isolated PRRSV in the lung tissues at 14 dpc from sacrificed piglets in a group (Ingelvac PRRS MLV+ QH-08) (Table 2). This is in agreement with earlier concern that isolated PRRSV in lung tissue might spread from those vaccinated pigs to non-vaccinated ones [26].

## 5. Conclusions

Out of the four commercial PRRSV vaccines in the efficacy experimental trial, only JXA1 and JXA1-R MLV vaccines exhibited complete protection against the QH-08 PRRSV challenge compared with type 2 Ingelvac PRRS MLV and CH-1α vaccines. In line with this, the mean rectal temperatures, lung lesion scores, and levels of virus load in serum and lung tissue were significantly lower in vaccinated and challenged pigs compared to the pigs in group 5. We conclude that the variation in protection capacity among these commercial vaccines might come from the difference in sequence identity of the all-open reading frames (ORF2-7, ORF5), particularly the coding sequences of ORF1a-1bs genes of the isolates/vaccine strains. In conclusion, we presented a novel insight in identifying the ORF1a-1b coding sequence as a potential fragment that could play a significant implication in the PRRSV vaccine’s partial or complete protection against the homologous virus challenge. Furthermore, sequence and phylogenetic analyses based on highly pathogenic PRRSV variant (QH-08 field isolate) from China in our previous study determined the clear genetic variations among the vaccine strains. These results might be useful for the development of an innovative PRRSV vaccine candidate in the future.

## Figures and Tables

**Table 1:** Amino acid sequence similarity between the vaccine strains and new type 2 PRRSV isolates (QH-08) based on whole-genome and coding sequences (ORF1a-1b and other six ORFS) originated from diverse epidemiological areas.

No.	Isolate	Genebank	Y	Whole	ORF1a-1b	ORF2	ORF3	ORF4	ORF5	ORF6	ORF7
1	CH-1a	AY032626	1998	40%	28.2%	96.1%	92.9%	97.2%	91.5%	96.8%	92.7%
2	JXA1	EF112445	2007	97.2%	96.3%	99.6%	98.4%	95.5%	97.5%	98.9%	99.2
3	QH-08	KU201579	2008	-	-	-	-	-	-	-	-
4	VR2332	EF536003	2007	37.9%	25.3%	91%	85.8%	89.9%	86.5%	96.6%	93.5

**Table 2:** Route of injection.

Groups		Vaccines Injected	Dose	Challenge Virus	Route of Injection
G1	5 pigs/each	Ingelvac PRRS MLV	2 mL/piglet	(QH-08 at (2 × 10^6^ TCID50) 2 mL/piglet	Intramuscularly
G2		CH-1α	2 mL/piglet		
G3		JXA1	2 mL/piglet		
G4		JXA1-R MLV	2 mL/piglet		
infected control	3 pigs/each	No vaccine	PBS-2 mL/piglet		
non-infected control				PBS-2 mL/piglet	

**Table 3:** The mean (±SD) compares macroscopic and microscopic lung lesion scores in different groups after QH-08 at 14 days post-challenge (dpc).

	Experimental Groups					
	G1	G2	G3	G4	Infected Control	Non-Infected Control
Vaccines	Ingelvac PRRS MLV	CH-1α	JXA1	JXA1-R MLV	No	No
challenge	QH-08					no
Macroscopic lung lesion score	55 ± 5.4 *	54 ± 4.8 *	32 ± 3.1	30 ± 2.8	82 ± 5 *	0/3
Microscopic lung lesion score	2.4 ± 0.3	2.3 ± 0.24	1.3 ± 0.2	1.1 ± 0.1	3.4 ± 5.4 *	0/3
Virus isolation	4/5 ^a^*	3/5 ^a^*	1/5	1/5	3/3 ^a^*	0/3

* denotes a significant difference in the macroscopic and microscopic lung lesion score between the groups from 2 to 14 dpc (*p* < 0.05). The letter "a" indicates the isolated PRRSV by QH-08 challenge from piglets, including dead pigs and necropsied pigs at a different time in the experiment period.

## Data Availability

The PRRSV sequences generated and analyzed during the current study are available in the GenBank, Accession: from AY032626, EF112445, EF536003, and KU201579. The other datasets used and/or analyzed during the present study are available from the corresponding author on reasonable request.

## Supplementary Materials

The following are available online at https://www.mdpi.com/article/10.3390/vaccines9091020/s1, Figure S1: Phylogenetic analysis of PRRSV Type 1&2 of 33 whole-genome sequences; the result showed that only six strains exhibited single homology with new isolate QH-08 as represented in the red box in the phylogenetic tree, title, Figure S2: Phylogenetic tree constructed from aligned amino acid sequences of ORF2s to ORF7s of Porcine productive and Respiratory Syndrome Virus (PRRSV) Type 1&2 with the reference sequence of QH-08 isolate by the neighbor-joining methods. Figure S3: This phylogenetic tree compares 32 different PRRSV vaccine and filed strains with reference strains (QH-08 isolate) (1991–2018) based on amino acid sequences of ORF5s. The closely related strains are marked with the red color inbox as indicated on the trees, Figure S4: This schematic diagram shows the overall experimental design in this study. On day 0, piglets were immunized with four vaccines and PBS. At 28 dpv, pigs in group-1 to group-5 were infected by QH-08. On 42th day, all piglets were necropsied and examined for macroscopic and microscopic lesions, Figure S5: Mutational ORF2 of QH-08, Figure S6: Mutational ORF3 of QH-08, Figure S7: Mutational ORF4 of QH-08, Figure S8: Mutational ORF5 of QH-08, Figure S9: Mutational ORF6 of QH-08, Figure S10: Mutational ORF7 of QH-08, Table S1: Comparison of genetic similarities based on the nucleotide sequence of whole-genome and ORF1a and 1b and ORF2-7 coding sequences of the QH-08 (China) reference PRRSV field isolates with 32 field and vaccine strains, originated from diversified epidemiological areas. Twelve out of these 33 PRRSV gene sequences are of Chinese origin.
