# Supplementary material for: Evaluation of Four Commercial Vaccines for the Protection of Piglets against the Highly Pathogenic Porcine Reproductive and Respiratory Syndrome Virus (hp-PRRSV) QH-08 Strain
Source: Vaccines (Basel). 2021 Sep 14;9(9):1020. doi: 10.3390/vaccines9091020 (PMC8471949; doi:10.3390/vaccines9091020)
Supplement: Supplementary file 1 [file vaccines-09-01020-s001.zip › vaccines-1346900-supplementary.pdf]

Supplementary material

Table S1. Comparison of genetic similarities based on the nucleotide sequence of whole-genome and ORF1a and 1b and ORF_{2-7} coding sequences of the QH-08 (China) reference PRRSV field isolates with 32 field and vaccine strains, originated from diversified epidemiological areas. Twelve out of these 33 PRRSV gene sequences are of Chinese origin.

No.	isolate	genebank	Y	Whole	ORF1a and 1b	ORF2	ORF3	ORF4	ORF5	ORF6	ORF7	Origin
1	CH-1a	AY032626	1998	40%	28.2%	96.1%	92.9%	97.2%	91.5%	96.8%	92.7%	
2	JXA1	EF112445	2007	97.2%	96.3%	99.6%	98.4%	95.5%	97.5%	98.9%	99.2	
3	HUN4	EF635006	2007	97%	96.1%	99.2%	98.8%	97.8%	98%	98.9%	99.2	
4	QH-08	KU201579	2008	-	-	256, -	254, -	-	-	174, -	-	
5	GM2	JN662424	2011	34.6%	19.9%	88.7%	88.2%	94.4%	81.5%	94.8%	88.6	
6	NVDC-CQ1-2012	KP771747	2012	96.8%	95.8%	99.6%	98.8%	97.8%	98%	98.9%	99.2	China
7	SD-A19	MF372560	2015	33.8%	24.9%	85.5%	79.9%	86%	16.1%	92.5%	90.2	
8	JL580	KR706343	2015	33.7%	19.9%	90.2%	97.2%	94.9%	84%	93.1%	89.4	
9	HK2	KF287133	2003	37.1%	24.3%	91.4%	85.4%	87.6%	83%	96%	93.5	
10	HK6	KF287135	2014	37%	24%	89.1%	84.3%	89.3%	86.5%	96%	92.7	
11	VR2332	EF536003	2007	37.9%	25.3%	91%	85.8%	89.9%	86.5%	96.6%	93.5	
12	NCV-13	KX192112	2016	33.9%	18.8%	85.2%	83.5%	88.8%	87.5%	93.1%	89.4	
13	MLV RespPRRS /Repro	AF159149	2000	36.5%	25.1%	91.8%	85.8%	89.9%	87.5%	96%	94.3	U.S.A
14	NADC30	JN654459	2008	34%	20.4%	85.9%	80.3%	88.2%	85.5%	92%	90.2	
15	CVK3-6	KP704287	2015	38.8%	26.5%	91.8%	87%	88.8%	86%	94.8%	91.9	Democratic People's Republic of Korea
16	PL97-1	AY585241	1997	38%	25.4%	92.2%	85.8%	89.9%	87.5%	10.9%	94.3	Korea
17	CA-2-MP110	KY363991	2016	34%	19.3%	85.2%	83.5%	89.9%	87.5%	93.1%	92.7	
18	HP/Thailand/19500LL/2010	KF735060	2013	95.7%	95.2%	98.4%	98.8%	99.4%	99%	98.8%	98.4	Thailand
19	01NP1.2	DQ056373	2005	38%	25.1%	92.2%	85.8%	89.9%	87.5%	93%	94.3	
20	SP	AF184212	1999	38%	25.6%	91.4%	85.4%	91.6%	91%	94.8%	94.3%	Singapore
21	PA8	AF176348	2002	37.8%	25%	91.8%	86.2%	88.8%	87%	96%	94.3%	Canada
22	DK-2011-030311-1	KC862577	2011	37.9%	25.3%	92.6%	85.8%	88.8%	88%	96.6%	94.3%	
23	DK-2003-2-3	KC862584	2003	37.3%	43.5%	90.6%	86.8%	90.4%	87.5%	96.6%	95.1	Denmark
24	VR-2332	AY150564	2002	38%	25.4%	92.6%	85.4%	89.9%	88%	96.6%	94.3	
25	PRRSV-2 Hungary 102 2012	KM514315	2012	33.9%	19.3%	87.1%	83.9%	88.2%	87%	93.7%	93.5	Hungary
26	TD1	KP998425	1997	37.3%	25.3%	91.8%	86.2%	90.4%	85%	94.3%	94.3	
27	MD001	KP998431	1991	35.9%	25.2%	91%	87%	91%	86%	96%	91.1	Taiwan
28	CH8V-J2	KP998426	2003	35.5%	22.2%	88.3%	84.6%	88.8%	85%	94.8%	94.3	
29	PRRSV/MZ/IND/1A/18	MK287894	2018	93.8%	94%	98%	97.2%	98.3%	96.5%	98.9%	96.7	India
30	BH58/10	JN626287	2010	95.9%	95.4%	98.4%	98.8%	99.4%	99%	98.9%	99.2	Laos

31	Aomori10-5	AB811789	2010	35.5%	22%	87.8%	82.6%	89.3%	87%	93.7%	85.2	Japan
32	EDRD-1	AB811785	1992	36.5%	23.1%	90.2%	84.6%	93.3%	89.5%	94.8%	93.5	
33	Lelystad virus	M96262	1993	27.4	9.3%	91%	23.6%	27.5%	5.5%	12.1%	15.4	Netherlands

The sequence alignment results showed that the amino acid identities of ORF1a and 1b among 33 sequences of species-2 strains ranges from (9.3-96.3 %) (JL580, JXA1). Higher sequence similarities (>93 %) among these strains based on the reference sequence observed recorded in JXA1, HUN4, NVDC-CQ1, 2012, HP/Thailand/19500LL/2010, PRRSV/MZ/IND/1A/18, and BH58/10. Similarly, sequence identities score based on the amino acid sequence of ORF range (16.1-99%) lower and higher in SD-A19 and H.P./Thailand/19500LL/2010), respectively. When compared nucleotide sequence identity of whole-genome of the genotype 1&2 based on QH-08 Chinese PRRSV reference strains, the isolates had (33.7-97.2%) closely and distantly related to SD-A19 and JXA1, respectively (Table 2).

The percentage of complete genome homology of these seven strains is 93.8-97.2% (PRRSV/MZ/IND/1A/18, JXA1), which is roughly similar to the percentage sequence identity based on ORF1a1b (94-96.3%, PRRSV/MZ/IND/1A/18 and JXA1 strain), and ORF5 sequence (96-99%, PRRSV/MZ/IND/1A/18, BH58/10 and H.P./Thailand19500LL/2010 strain) (Table 2). The ORF2-ORF7 of 7 strains on the same lineage has a higher 98% amino acid sequence similarity than other strains.

Phylogenetic analysis of whole-genome sequence showed that only seven strains exhibited single homology with QH-08 isolate (Figure 2, red box). Meanwhile, ORF2-ORF7 and ORF 5 aligned sequence analysis showed six strains formed the single homology with QH-08 (Figures 2 and 3, red box). The homology of these seven strains is 93.8-97.2% (PRRSV/MZ/IND/1A/18, JXA1) is directly related to the ORF1a and 1b of 7 genotypes-2 ranges from 94-96.3 % including of JXA1、HUN4、QH-08、NVDC-CQ1-2012 、 PRRSV/MZ/IND/1A/18 、 H.P./Thailand19500LL/2010 and BH58/10. So, based on these facts, we investigated that the gap of ORF1a and 1b homology caused the difference of the whole sequence of PRRSV and may reveal partial protection of the PRRS vaccine when some homologous virus challenged.

On the other hand, a higher score of sequence identity recorded in ORF6 is 92-98.9% (HENAN-XINX, HUN4), ORF7 is 88.6-99.2% (GM2, HUN4), ORF4 is 88.2-99.4% (NADC30, H.P./Thailand/19500LL/2010), ORF2 is 85.2-99.6% (CA-2-MP110, JXA1), ORF3 is 80.3-98.8% (NADC30, HUN4) as detailed in (Table 2). Briefly, we found that the ORF6 sequences (92%–98.9%) of the strains of the Chinese mainland showed distinctive conservation compared to the other genes, followed by ORF7, ORF4, ORF2, ORF3, and ORF5. ORF7, ORF2, and ORF5s genes exist in insertion and deletion, such as Aomori10-5 and JXA1.

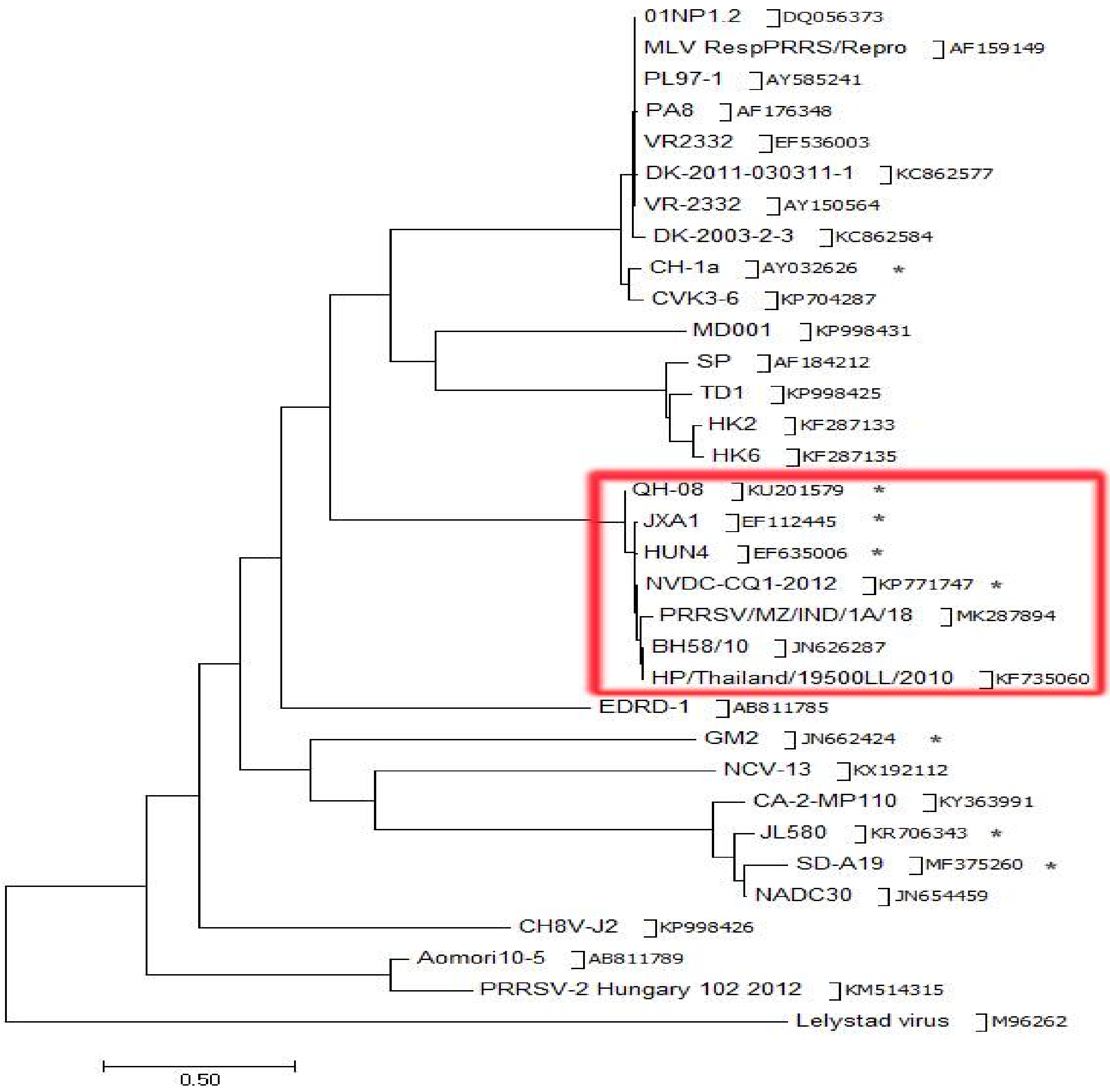

Figure S1. Phylogenetic analysis of PRRSV Type 1&2 of 33 whole-genome sequences; the result showed that only six strains exhibited single homology with new isolate QH-08 as represented in the red box in the phylogenetic tree.

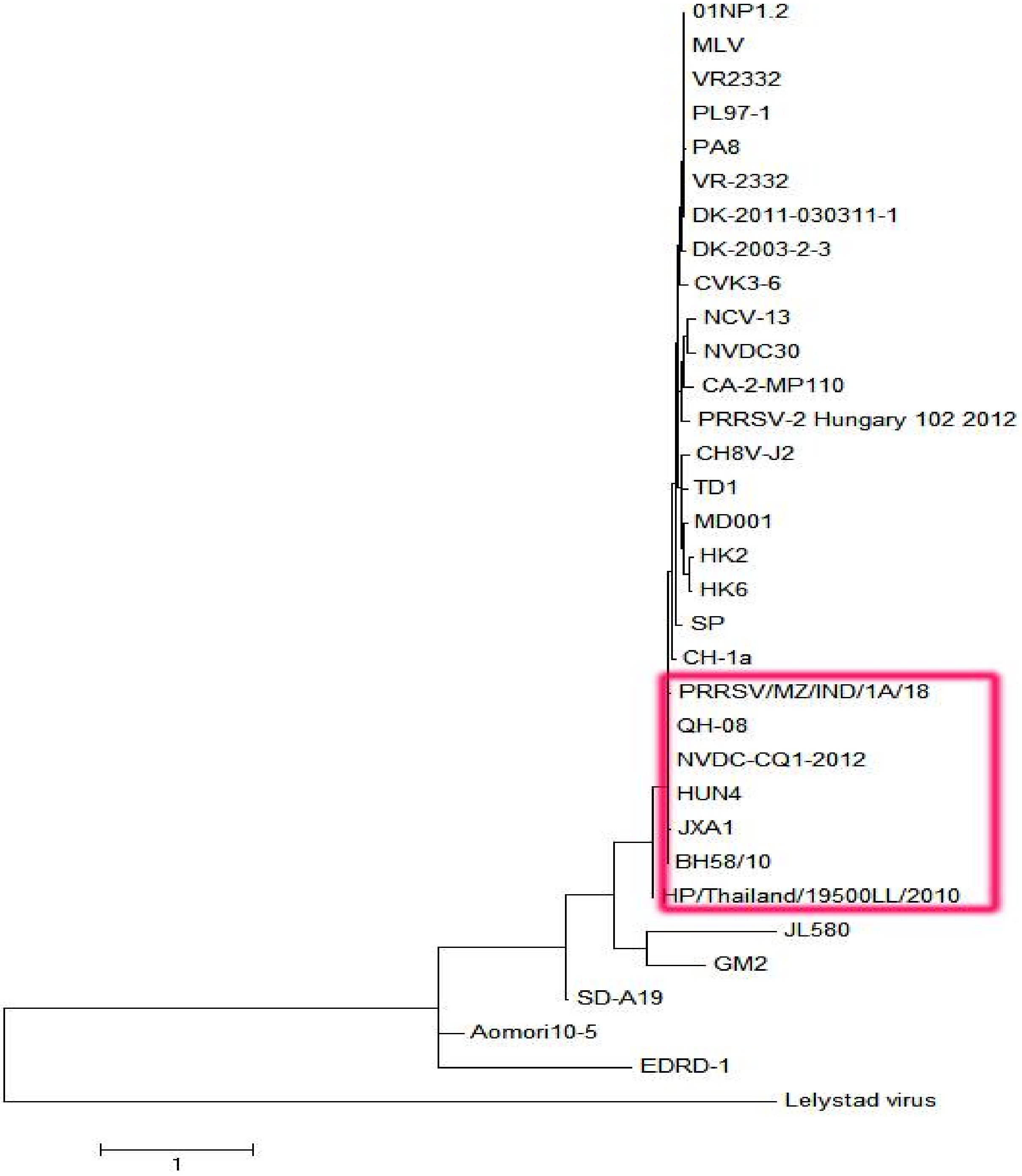

Figure S2. Phylogenetic tree constructed from aligned amino acid sequences of ORF2s to ORF7s of Porcine productive and Respiratory Syndrome Virus (PRRSV) Type 1&2 with the reference sequence of QH-08 isolate by the neighbor-joining methods.

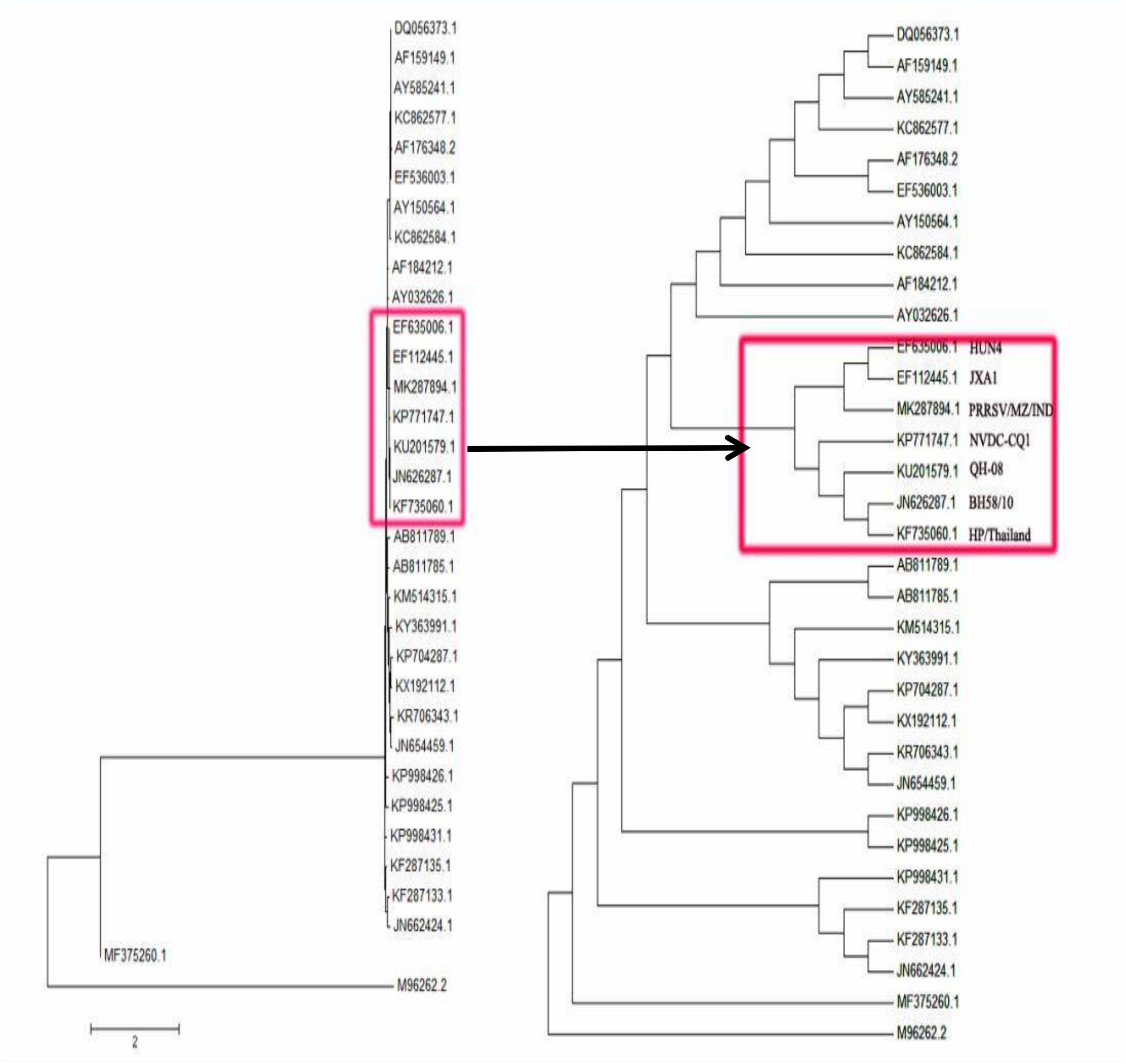

Figure S3. This phylogenetic tree compares 32 different PRRSV vaccine and filed strains with reference strains (QH-08 isolate) (1991– 2018) based on amino acid sequences of ORF5s. The closely related strains are marked with the red color inbox as indicated on the trees.

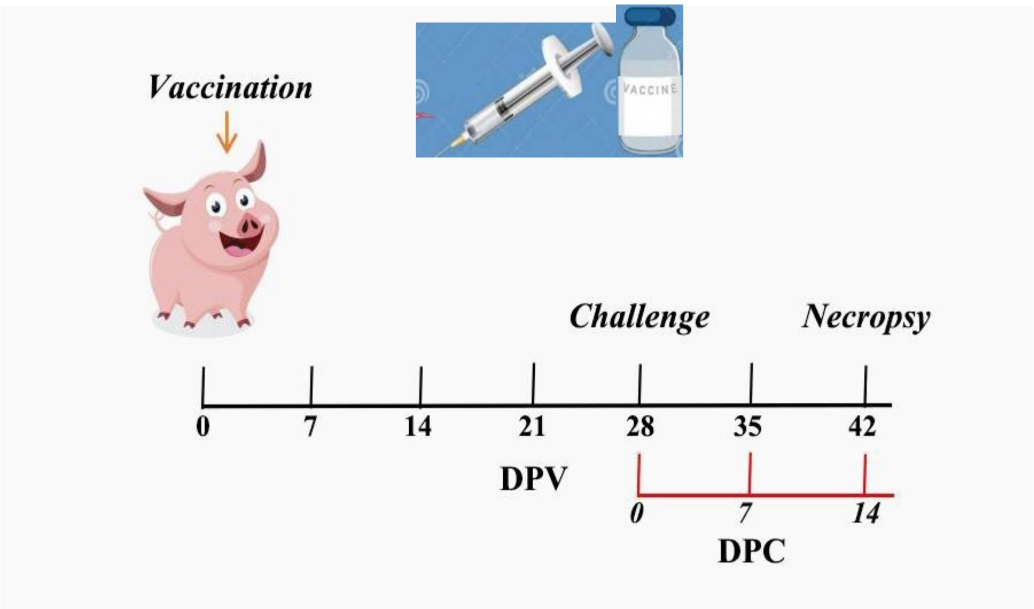

Figure S4. This schematic diagram shows the overall experimental design in this study. On day 0, piglets were immunized with four vaccines and PBS. At 28 dpv, pigs in group-1 to group-5 were infected by QH-08. On 42th day, all piglets were necropsied and examined for macroscopic and microscopic lesions.

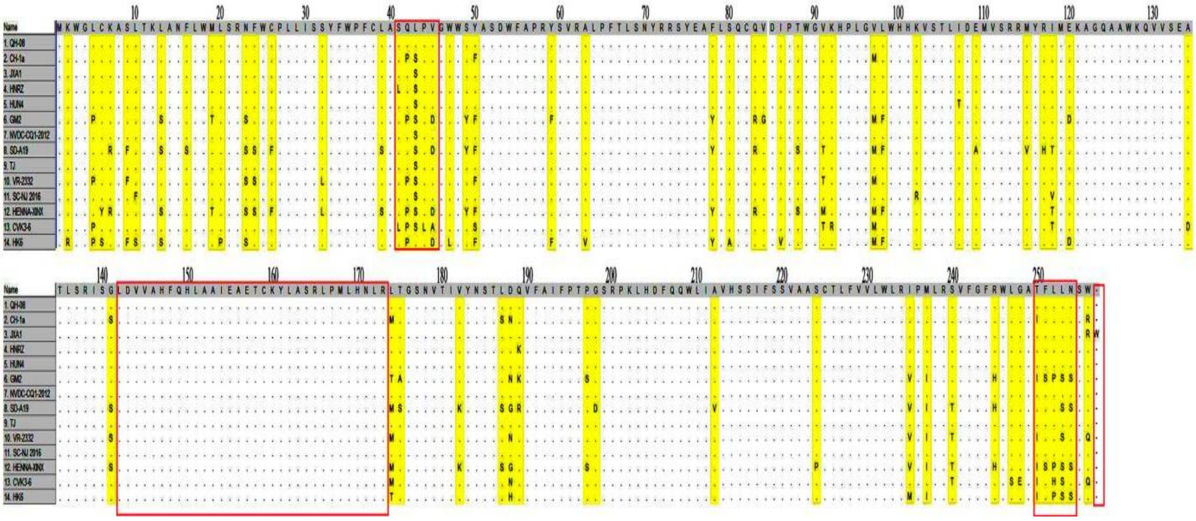

Figure S5. Mutational ORF2 of QH-08.

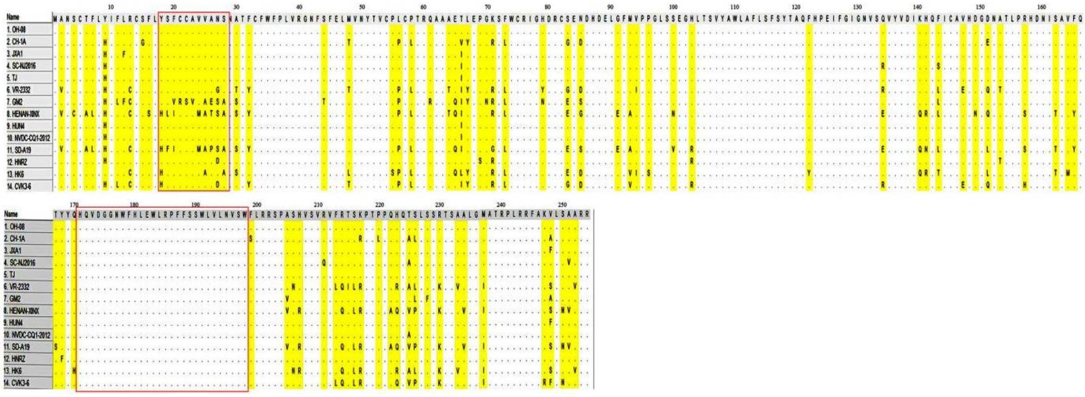

Figure S6. Mutational ORF3 of QH-08.

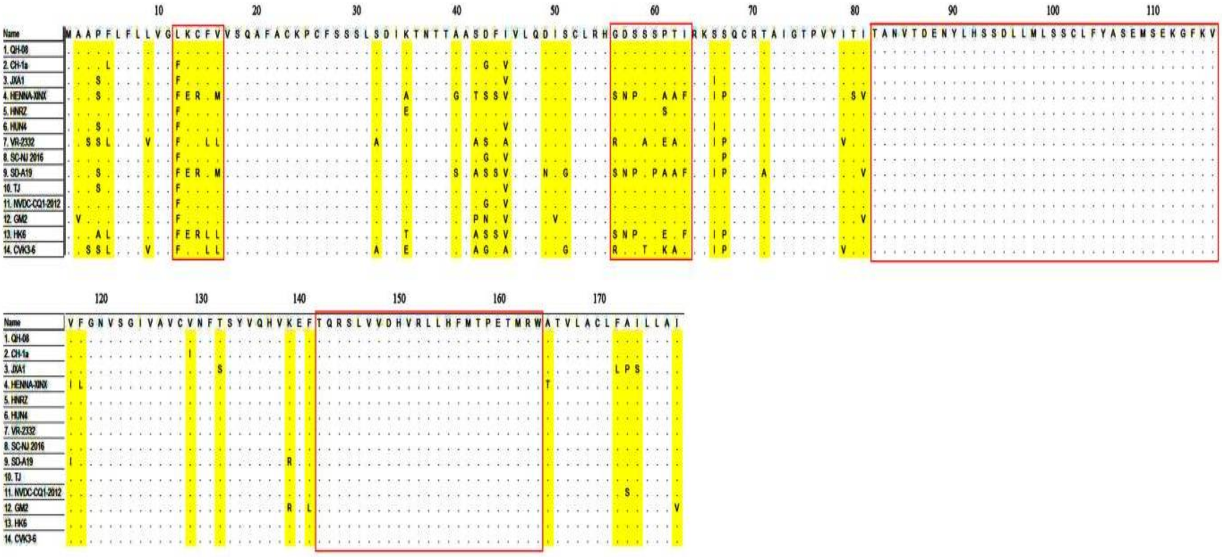

Figure S7. Mutational ORF4 of QH-08.

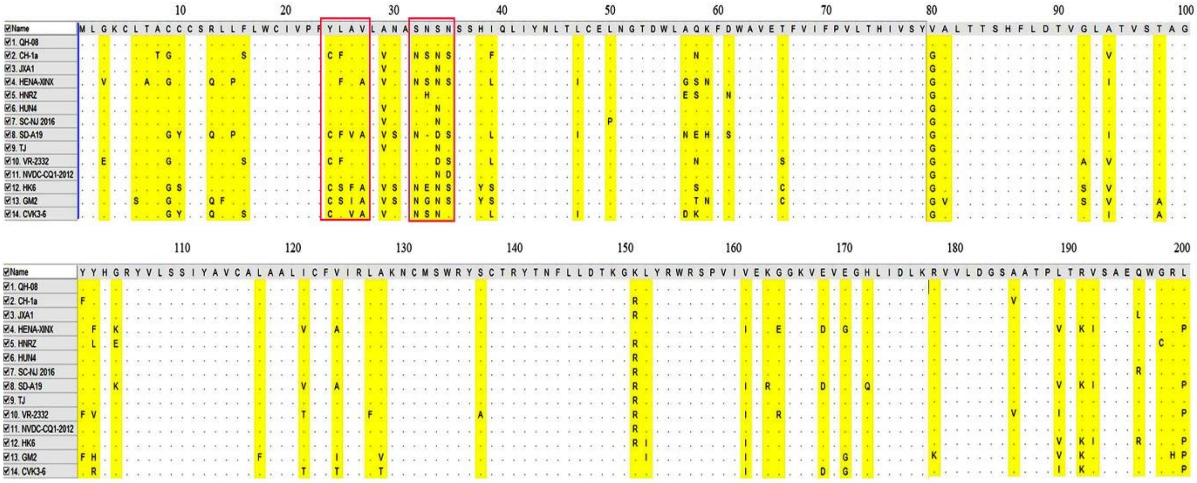

Figure S8. Mutational ORF5 of QH-08.

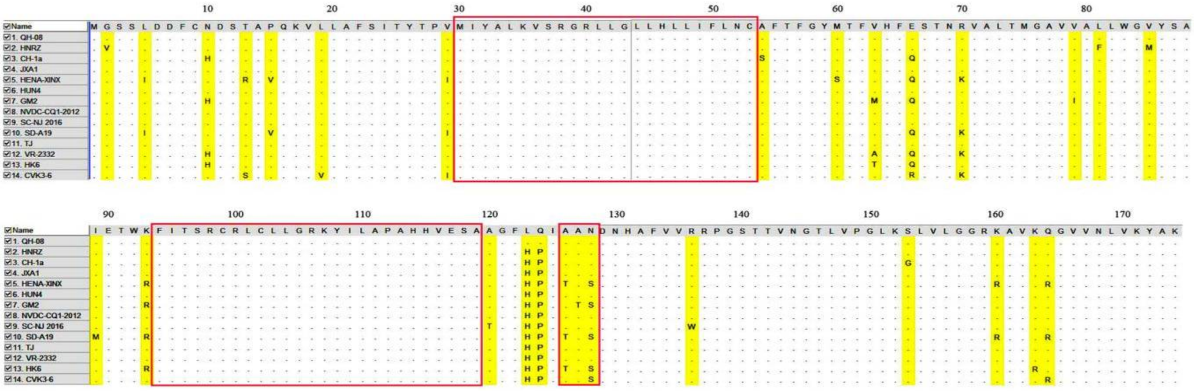

Figure S9. Mutational ORF6 of QH-08.

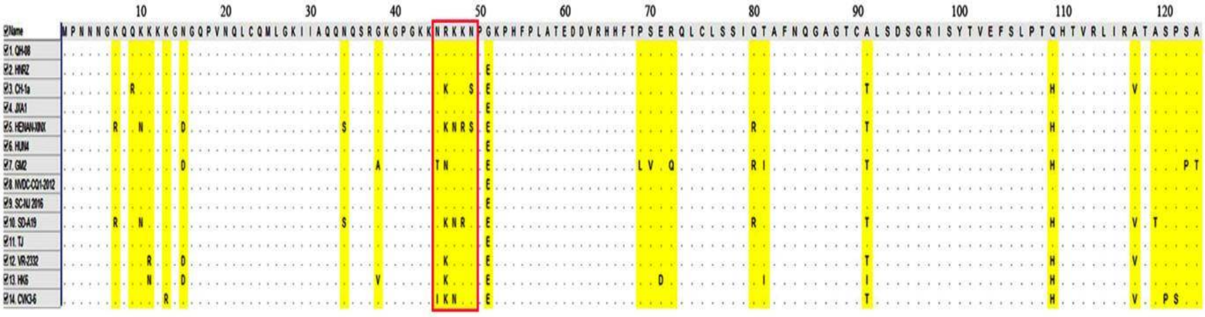

Figure S10. Mutational ORF7 of QH-08.
